# Procedures for health data linkage: applications in health surveillance

**DOI:** 10.1590/S2237-96222022000300004

**Published:** 2022-10-10

**Authors:** Klauss Kleydmann Sabino Garcia, Cristiano Barreto de Miranda, Flávia Nogueira e Ferreira de Sousa

**Affiliations:** 1 Universidade de Brasília, Núcleo de Medicina Tropical, Brasília, DF, Brazil; 2 Universidade de São Paulo, Faculdade de Saúde Pública, São Paulo, SP, Brazil

**Keywords:** Data Analysis, Epidemiology, Public Health, Public Health Surveillance

## Abstract

**Objective:**

To present a standardized methodology for linking different public health databases.

**Methods:**

This was a methodological review article specifically describing data processing procedures for deterministic linkage between structured databases. It instructs on how to: treat data, select linkage keys, and link databases using two databases simulated in R software.

**Results:**

The commands used for the deterministic linkage of the inner_join type were presented. The linkage process resulted in a database with 40,108 pairs using only the “Name” key. Adding the second key, “Name of mother”, the resulted dropped to 112 pairs. By adding the third key, “Date of birth”, only two pairs were identified.

**Conclusion:**

Database linkage and its analysis are valid and valuable tools for health services in supporting health surveillance actions.

**Study contributions**

**Main results**

The main result of this work is the standardized method model for linking data from different public health information systems.

**Implications for services**

This work will enable replication of standardized data linkage models, using a technical-scientific basis, for health situation analyses.

**Perspectives**

It is expected that the procedures presented will be used in their entirety or as models for data linkage processes in health services and research or educational institutions, for the improvement of epidemiological analyses.

## INTRODUCTION

Health surveillance is defined as a continuous and systematic process of data collection, consolidation and analysis, as well as dissemination of information on health-related events, aiming at the planning and implementation of public health measures that include regulation, intervention and action on health determinants, for the protection and promotion of the population’s health, and the prevention and control of risks, health conditions and diseases.^1^

Integration of health information systems (HIS) is an important strategy described in the Brazilian National Health Surveillance Policy, in order to contribute to the improvement and consolidation of health surveillance management, especially in activities involving planning, monitoring and evaluation of surveillance actions, in a timely manner.^1^ HIS are technological tools that assist in the processing of data collected in health services and elsewhere, generating useful information for understanding problems and informing decision making within the scope of health policies and care.^2^ ^,^ ^3^

In Brazil, the Ministry of Health is responsible for the management of national HIS databases. Among the various HIS, the most commonly used are: the Mortality Information System (SIM), the Live Birth Information System (SINASC), the Notifiable Health Conditions Information System (SINAN), the National Health System Hospital Information System (SIH/SUS), the Epidemiological Surveillance Information System (SIVEP) and, recently, the ‘e-SUS Notifica’ system. Despite the availability and improvement of several HIS in recent decades, interoperability between them does not yet occur in the health surveillance sectors.^2^

In view of the need to work with qualified information, which ensures integration between the different HIS, database linkage presents itself as a viable strategy, with the purpose of materializing this connection of different sources of information.^4^ The linkage technique, which is of relatively low operational cost, allows recovery of incomplete or inconsistent records and thus improves the completeness and reliability of the information provided by HIS.^5^

Although previous studies show the advantages of using linkage to analyze the quality of several HIS,^6^ ^-^ ^9^ there is a scarcity of methodological studies that present the procedures necessary to perform this technique. This manuscript is therefore justified by the importance of disseminating methodologies used in health surveillance, in the methodological standardization of data linkage, and in providing analysis models that can be adapted to different realities and areas of knowledge regarding health. 

The objective of this work was to present a standardized methodology for linking different public health databases.

## METHODS

This was a methodological review article, on the linking of databases from different information systems, considering technical procedures performed within the scope of health surveillance of the Brazilian Ministry of Health. The method provided here was developed for use in R software, from simulated databases using hypothetical information about the health service user’s name, their mother’s name and their date of birth.

The methods described below are replicable for any data from different HIS, and are focused on the deterministic linking process, which performs classification of possible data pairs based on matching rules intended to match identical records.^10^

###  Preparation of the databases 

Before starting the data linkage process, it is important to define the variables to be used to analyze the linked data. Defining them will inform the data treatment process, since when working with very large databases, it may be necessary to reduce the database size so that the computer can process the data more easily, thus avoiding performance problems. In addition, the R software may malfunction due to the limitation of the program’s internal memory when large databases are being used. It is therefore recommendable to work objectively, using only variables of interest, in order to achieve better linkage process efficiency.

### *Linkage with* R *Software*

Use of R software is suggested for the process of developing the data treatment and linkage script.

The following packages will need to be installed: lubridate^11^ (enables treatment of “date” type variables, e.g., “Date of notification”), *abjutils* ^12^ (enables removal of accents on letters) and *tidyverse*.^13^ The “*randomNames*”^14^ package and function are suggested for simulating nominal data.

The *tidyverse* ^13^ package includes other packages, more frequently used in data treatment and analysis, such as: *ggplot2* (enables production of graphics), *dplyr* (data manipulation), *tidyr* (data organization), *readr* (reading .csv files), *purrr* (enables working with functions and vectors), *tibble* (reading databases in .txt text format), *stringr* (enables working with nominal variables) and *forcats* (treatment of categorical variables) - command: install.packages(“*tidyverse*”). If the software is closed, the library function must be used to load the packages of interest that have already been installed - command: library(*tidyverse*).

###  Data treatment 

It is important to replicate the steps described below following the same order as given in these step-by-step instructions, in order to avoid errors. 

Initially, the working directory (file folder) must be defined using the setwd function. The databases to be linked must be included in this directory - command: setwd(“C:/Users/User/Desktop/Work folder”). 

To import the databases into the R environment, we suggest using the read.csv2 function - which imports .csv files with a “;” separator instead of a “,” separator. For databases in text format (txt), the *read.table* function should be used, setting the first row as a variable header, the cell separator as “;” and the decimal marker as “,”. If .xls or .xlsx files are used, the readxl::read_excel(“file.xlsx”) command must be used. In the case of .dbf or .dbc files,^15^ which are common for the SINAN HIS data, the “foreign”^16^ and “read.dbc” packages need to be installed,^17^ and the read.dbf(“file.dbf”) and read.dbc::read.dbc(“file.dbc”) commands need to be used, respectively.

We suggest that the first imported database be attributed to an R storage object, called “Database_1”, and the second database to another object, called “Database_2” - command: Database_1 <- read.csv2(“Database 1.csv”); or read.table(“Database_1.txt”, header = TRUE, sep = “;”, dec = “,”).

We suggest that the variables that will not used be removed from each database, using the command “Database_1$variable <- NULL”. If there is a large amount of variables to be removed, instead of excluding them we suggest selecting the variables of interest, using the select function and attributing this new set of variables to the same object previously created - e.g.: Database_1 <- select(Database_1, variable1, variable3, variable5).

It is essential to keep the variables that will be used as keys for linkage, together with those that will be analyzed. We suggest using the variables referring to the person’s name, their CPF (taxpayer identification number), their date of birth and their mother’s name, because they are the most frequently used keys in the scientific literature. Although it is uncommon in HIS databases, the “CPF” variable can be taken to be a variable that has periods and hyphens in its structure. In this way, the CPF can be considered to be a character type variable, and to use it as a numeric variable (numeric or integer), these periods and hyphens need to be removed from the observations. Moreover, the CPF can be filled in in different ways on these HIS notification forms, so that it may or may not contain periods and hyphens. Given the lack of standardization in filling in this variable, we suggest removing any periods and hyphens from it in order to achieve the best use of this key.^18^ ^-^ ^20^

In order to standardize the “name” and “CPF” variables, we suggest that they be used as character type variables (text). If these variables need to be converted, the following command should be used: as.character(Database_1$variable_1). In the case of the CPF, the periods “.” and hyphens “-” should be removed (command: Database_1$CPF <- str_replace_all(Database_1$CPF, “\\.|-”, “”), adding zero “0” digits to the left of the text until it has 11 digits (command: Database_1$CPF <- str_pad(RAIS Database_1$CPF, 11, pad=”0” ).

 With regard to the “date of birth” variable (*data do nascimento* in Portuguese), the user must check whether it appears in “Date” format in the R environment. The type of variable can be checked using the class function - command: class(Database_1$Data_nascimento).

In R dates are shown in the following format: 2020-12-31 (year-month-day). If the variable is not in this “Date” format, it can be converted to it using *lubridate* ^11^ package functions, using the following command, for example: Database_1$Data_nascimento <- as.Date(Database_1$Data_nascimento). Other variations of this command can be used, such as dmy (in the case of dates written in day-month-year format) or ymd (for dates in year-month-day format).

Regarding “Name” variables (*Nome* in Portuguese), first of all any letters with accents should be replaced with letters without accents, e.g.: replace “Á” with “A”, “Õ” with “O” and so on. Elements that join surnames can also be removed, such as “E”, “A”, “DA”, “DE”, “DO”, “DAS”, “DES” and “DOS”, e.g.: “MARIA DOS ANJOS” becomes “MARIA ANJOS”. We suggest that the letters of names be turned into capital letters, as the program distinguishes between uppercase and lowercase letters. The command for this is: toupper(Database_1$Nome). The function used to replace letters is str_replace_all - command: Database_1$Nome <- str_replace_all(Database_1$Nome,” DAS “, “ “); or, to replace at the same time all elements that join surnames, str_replace_all(Database_1$Nome,”\ | DA |,|;|:|-| DE | E | DO | DAS | DOS | “, “ “ ).

Once these replacements have been performed, we suggest that blank spaces between names be removed, as there may be double spaces between names, and this hinders deterministic linkage processes - command: Database_1$Nome <- str_replace_all(Database_1$Nome,” “, “” ).

This process of standardizing the person’s name, CPF, date of birth and mother’s name variables takes into consideration the possibility of small inconsistencies in the filling in of this information by health workers, on the different information systems.

Once this information has been cleaned and standardized, the database can be checked for duplicated records. The *distinct* function can be used to exclude duplicated records; however, whether or not duplicated records are to be excluded needs to be discussed beforehand, considering the objective of the result of the data linkage.

The number of keys to be used in the process must also be established prior to linkage. The combination of different keys, such as the “Nome_paciente” (patient name) key combined with the “Data_nascimento” (date of birth) key, will result in a more specific linkage process, aiming at a greater number of true positive results. Using only one key, such as “Nome_paciente”, will provide more sensitive results, with more false positive pairs. Furthermore, R software performs combinatory analyses for possible pairs; therefore, if there are five people with the name “MARIA DOS ANJOS” in each database, the software will return a linked database with 25 results. In view of this, we suggest that at least two key variables be used in linkage.

The decision to use more than two keys for linkage should be based on database quality analysis, since the deterministic process can be affected by data that has been input incorrectly; for example, when different birth dates are found but, through manual analysis, the researcher can identify that they are the same, but were input with a typing error. Thus, it may be interesting to use more sensitive methods, as long as this also involves manual cleaning of possible true negative pairs (Box 1).

**Box 1:** - Examples of key variables used for linkage, and possible results

Database 1			Database 2			Result	Verdict
Patient_name^a^	Date_of_ birth^a^	Name_of_mother	Patient_name^a^	Date_of_ birth^a^	Name_of_mother		
MariaAnjos	27-09-1994	LurdesSilva	MariaAnjos	27-09-1994	LurdesSilva	Matched	True positive
MariaAnjos	27-09-1994	LurdesSilva	MariaAnjos	27-09-1994	AnaCleide	Matched	False positive
MariaAnjos	27-09-1994	LurdesSilva	MariaAnjos	13-03-2001	MariaDores	Non-matched	True positive
MariaAnjos	27-09-1994	LurdesSilva	MariaAnjos	27-09-1894	LurdesSilva	Non-matched	False positive

a) Field used as linkage key.

###  Data linkage 

Deterministic linking identifies pairs of matching records, based on a given set of rules.^21^ It is indicated when the databases to be worked on have a common identifying variable or a set of variables that have been filled in correctly.^10^ ^,^ ^22^ ^,^ ^23^

**Figure 1:**
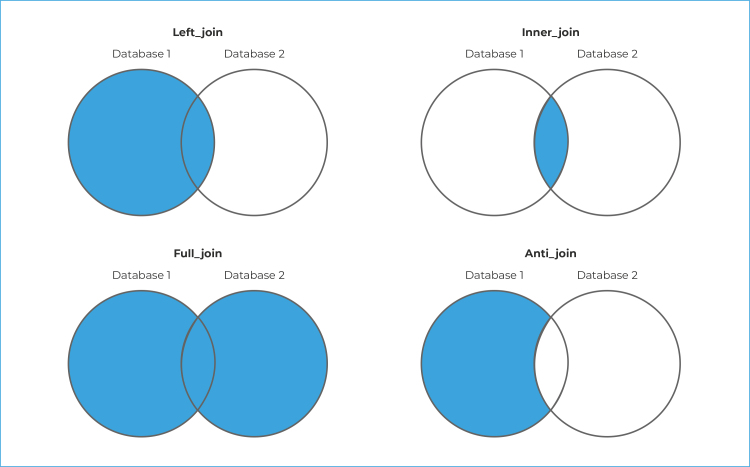
- Representation of types of database joining, by R software functions

Using R software it is possible to perform different forms of linkage. The most frequent forms, described below, are exemplified in Box 2. The functions to be used are “left_join”, “*inner_join*”, “*full_join*” and “semi_join”.^24^ The *left_join* function feeds Database 1 (Database_1) with information from Database 2 (Database_2). The *inner_join* function only returns information with keys common to both databases, i.e. only matching records. The *full_join* function brings both databases together, with no loss of information. The *anti_join* returns Database 1 records that were not matched with Database_2 (Figure 1). 

In order to facilitate the operation of the functions described previously, it is recommended that the key variables for linkage be given exactly the same name, otherwise the functions will need the different key names to be specified.

After linkage is complete, the database is ready to be explored and analyzed. The main packages for analyzing this data will have already been loaded indirectly, when the *tidyverse* ^13^ package was loaded.

###  Simulation of the deterministic linkage process 

Two datasets were created to simulate deterministic linkage using the “randomNames” function of the “randomNames” package.^14^ Database_1 was created with 10,000 observations and Database_2 with 500,000 observations. The following simulated variables were included in the databases: “Nome” (name and surname), “Nome da mãe” (mother’s name, only her first name), “Data de nascimento” (date of birth), “Nome do Banco” (database name) and two further test variables (test X, test Y, Test W, and Test Z), totaling six variables in each database. The R script for creating simulated databases is available in the supplementary material (https://github.com/kleydmann/Modelo_Vinculacao_deterministica.git).

## PERFORMING LINKAGE

Data script:

Installing and loading the packages needed:

install.packages(“tidyverse”)

install.packages(“lubridate”)

install.packages(“abjutils”)

library(tidyverse)

library(lubridate)

library(abjutils)

Defining the directory:

setwd(“C:/Users/User/Desktop/Work folder”)

Importing the databases to the R environment:

Database_1 <- read.csv2(“Database_1.csv”)

Database_2 <- read.csv2(“Database_2.csv”)

Excluding variables, if necessary:

Database_1$X_Y <- NULL

Database_2$X_Z <- NULL

Selecting the variables of interest, if necessary:

Database_1 <- select( Database_1, Nome, Nome_mae, DT_NASC, Database, variavel_X)

Database_2 <- select( Database_2, Nome, Nome_mae, DT_NASC, Banco, variavel_Z)

Following data treatment, Database_1 and Database_2 were comprised of 10,000 and 500,000 observations, respectively, each with five variables. 

Checking the coding of the key variables:

> class(Database_1$Nome)

[1] “character”

> class(Database_1$Nome_mae)

[1] “character”

> class(Database_1$DT_NASC)

[1] “Date”

> class(Database_2$Nome)

[1] “character”

> class(Database_2$Nome_mae)

[1] “character”

> class(Database_2$DT_NASC)

[1] “Date”

Converting the “Data de nascimento” (date of birth) variable format:

Database_1$DT_NASC <- ymd(Database_1$DT_NASC)

Database_2$DT_NASC <- ymd(Database_2$DT_NASC)

Converting letters into uppercase:

Database_1$Nome <- toupper(Database_1$Nome)

Database_1$Nome_mae <- toupper(Database_1$Nome_mae)

Database_2$Nome <- toupper(Database_2$Nome)

Database_2$Nome_mae <- toupper(Database_2$Nome_mae)

Treating the “Nome” (name) and “Nome_mae” (mother’s name) variables of Database_1 and Database_2 (removal of joining words, prepositions, accents and double spaces):

Database_1$Nome <- str_replace_all(Database_1$Nome,”\ | DA |,|;|:|-| DE | E | DO | DAS | DOS | “, “ “ )

Database_1$Nome <- rm_accent(Database_1$Nome)

Database_1$Nome_mae <- str_replace_all(Database_1$Nome_mae,”\ | DA |,|;|:|-| DE | E | DO | DAS | DOS | “, “ “ )

Database_1$Nome_mae <- rm_accent(Database_1$Nome_mae)

Database_2$Nome <- str_replace_all(Database_2$Nome,”\ | DA |,|;|:|-| DE | E | DO | DAS | DOS | “, “ “ )

Database_2$Nome <- rm_accent(Database_2$Nome)

Database_2$Nome_mae <- str_replace_all(Database_2$Nome_mae,”\ | DA |,|;|:|-| DE | E | DO | DAS | DOS | “, “ “ )

Database_2$Nome_mae <- rm_accent(Database_2$Nome_mae)

Removing blank spaces:

Database_1$Nome <- str_replace_all(Database_1$Nome,” “, “” )

Database_1$Nome_mae <- str_replace_all(Database_1$Nome_mae,” “, “” )

Database_2$Nome <- str_replace_all(Database_2$Nome,” “, “” )

Database_2$Nome_mae <- str_replace_all(Database_2$Nome_mae,” “, “” )

Deterministic linkage using the *Inner_Join* method (only records common to both databases)

Using 1 key:

Linked_database_1_key <- inner_join(Database_1,Database_2, by = “Nome”)

Using 2 keys:

Linked_database_2_keys <- inner_join(Database_1,Database_2, by = c(“Nome”,”Nome_mae”))

Using 3 keys:

Linked_database_3_keys <- inner_join(Database_1,Database_2, by = c(“Nome”,”Nome_mae”,”DT_NASC”))

The *inner_join* deterministic linkage process using just one key variable (“Nome”) (name) resulted in a database with 40,108 possible pairs and nine variables. Using just one key resulted in a number of possible pairs that was greater than the total number of records held on Database_1. This was due to the combinatory analyses that the R software performs during linkage.

**Box 2:** - Examples of R software data linkage functions

Method	Number of keys	Functions
left_join	1	left_join(Database_1, Database_2, by = " Nome_paciente ")
	2	left_join(Database_1, Database_2, by = c("Nome_paciente ", "Data.de.Nascimento"))
	3	left_join(Database_1, Database_2, by = c("Nome_paciente ", "Data.de.Nascimento", "nome.da.mae"))
inner_join	1	inner_join(Database_1, Database_2, by = "Nome_paciente ")
	2	inner_join(Database_1, Database_2, by = c("Nome_paciente ", "Data.de.Nascimento"))
	3	inner_join(Database_1, Database_2, by = c("Nome_paciente ", "Data.de.Nascimento", "nome.da.mae"))
full_join	1	full_join(Database_1, Database_2, by = "Nome_paciente ")
	2	full_join(Database_1, Database_2, by = c("Nome_paciente ", "Data.de.Nascimento"))
	3	full_join(Database_1, Database_2, by = c("Nome_paciente ", "Data.de.Nascimento", "nome.da.mae"))
anti_join	1	anti_join(Database_1, Database_2, by = "Nome_paciente ")
	2	anti_join(Database_1, Database_2, by = c("Nome_paciente ", "Data.de.Nascimento"))
	3	anti_join(Database_1, Database_2, by = c("Nome_paciente ", "Data.de.Nascimento", "nome.da.mae"))

When two key variables were used (“Nome” and “Nome da mãe”) (name and mother’s name), 112 pairs were found (1.12%). When the third key variable was added, “Data de nascimento” (date of birth), only two (0.02%) pairs were identified.

## DISCUSSION

Although database linkage has been described in several studies^25^ ^-^ ^28^ as a relevant technique for improving the quality of health information, its use is not yet widespread in health surveillance settings.

The main advantages of linkage for health services include (i) the possibility of improving the information coming from information systems, with recovery of incomplete information, and (ii) identification of errors in data completion. Maia, Souza & Mendes^25^ found that the linkage technique contributed to improving the quality of infant mortality data in five Brazilian cities, with 92% recovery of incomplete fields on the SIM and SINASC systems. Tuberculosis data in the municipality of Rio de Janeiro were improved, with a reduction of inconsistencies in the database after linkage using data from the SIM and AIDS (SINAN) databases.^28^

As for the methodological approach of this work, the probabilistic approach was not used because it requires the definition of matching rules and cutoff points for similarity indices, thus making it necessary to develop a specific study for such techniques.^23^

The linkage strategies discussed in this study tend to have a low number of true matches identified when using many key variables in the matching process. Therefore, it might be interesting to use phonetic codes combined with other variables, this being a possible alternative for increasing the sensitivity of the method.

Notwithstanding, it is essential to define sets of rules that allow the use of linkage keys that are precise, stable over time and common to the different databases of interest.^20^

The four different functions used for linkage in this study enable different ways of exploring the data after linkage. The most used methods are *left_join* and *inner_join*, as they take the first database as a reference and the second as a source of new information. Thus, the *left_join* method can be used, for example, to complement information regarding “Occupation”, a variable that is not found on all the SINAN system notification forms, whilst also being present in some Ministry of Labor and Welfare information systems. The *inner_join* method can be used, for example, to match information from SINAN system case notifications with death notifications held on the SIM system, allowing for specific analysis of cases that progressed to death.

Prior cleaning of the data to be matched allows an increase in overall accuracy of the linkage algorithm employed, since some databases contain poor quality data. 

It is important to mention that the level of operationalization and replicability used in procedures described in this paper is extremely simple. However, depending on the complexity of the linkage that is being planned, other packages, such as “RecordLinkage”,^29^ and other methods may be more appropriate.

In any case, attention needs to be paid to matchings between databases that contain duplicate records or different records for the same person, because the functions described in this paper result in different possibilities of combinatorial analysis in the event of identical records.

The limitations described here, regarding the general linkage method and techniques, are mainly related to the possibility of systematic errors arising from the use of secondary databases, since the use of large databases makes the task of manually checking for possible false-positive pairs burdensome. Furthermore, the limitation of R software in managing internal memory is an aspect that can hinder the carrying out of linkage. Therefore, it is essential to discuss the linking process in advance, in order to reduce the possibility of selection biases and software operational problems.

The quality of the linked database is dependent on the quality of the original databases. Therefore, it is important that a prior assessment of the quality of the data is made in order to identify the shortcomings and limitations of each database and, if possible, correct them before linkage. This is an important step in analytical preprocessing in data analysis.

## FINAL CONSIDERATIONS

Linked database analyses show themselves to be a valid and useful tool for health services, especially those at the state and federal levels of government.

These methods enable descriptive studies and have the potential to support analytical studies, as well as to provide information that can contribute to the development of strategic health actions and the promotion of public health policies aimed at more vulnerable populations. Incorporation of the linkage method in the routine of health services can be a tool to be used to contribute to the implementation of more appropriate actions, aiming at improving health surveillance.

Finally, the very need to implement data linkage processes highlights the fragility of interoperability between government information systems. This shortcoming needs to be addressed, in order to promote better integration between information systems, both in health services and also in other government areas.
